# Vitellointestinal Duct Anomalies in Infancy

**DOI:** 10.21699/jns.v5i3.351

**Published:** 2016-07-03

**Authors:** Yogender Singh Kadian, Anjali Verma, Kamal Nain Rattan, Pardeep Kajal

**Affiliations:** 1 Department of Pediatric Surgery, Pt. BD Sharma PGIMS, Rohtak, Haryana, India; 2 Department of Pediatrics, Pt. BD Sharma PGIMS, Rohtak, Haryana, India

**Keywords:** Vitellointestinal duct, Umbilicus, Infancy

## Abstract

Background: Vitellointestinal duct (VID) or omphalomesenteric duct anomalies are secondary to the persistence of the embryonic vitelline duct, which normally obliterates by weeks 5–9 of intrauterine life.

Methods: This is a retrospective analysis of a total of 16 patients of symptomatic remnants of vitellointestinal duct from period of Jan 2009 to May 2013.

Results: Male to female ratio (M:F) was 4.3:1 and mean age of presentation was 2 months and their mode of presentation was: patent VID in 9 (56.25%) patients, umbilical cyst in 2(12.25%), umbilical granuloma in 2 (12.25%), and Meckel diverticulum as content of hernia sac in obstructed umbilical hernia in 1 (6.25%) patient. Two patients with umbilical fistula had severe electrolyte disturbance and died without surgical intervention.

Conclusion: Persistent VID may have varied presentations in infancy. High output umbilical fistula and excessive bowel prolapse demand urgent surgical intervention to avoid morbidity and mortality.

## INTRODUCTION

The omphalomesenteric duct (OMD) is an embryonic structure, which connects the yolk sac to the midgut and failure of its resorption results in various anomalies including Meckel's diverticulum, patent vitelline duct, fibrous band, sinus tract, umbilical polyp and cyst, enteric fistula with ileal intussusception prolapsing over the umbilicus or hemorrhagic umbilical mass. These anomalies occur in approximately 2% of the population and may remain silent throughout life, or may present incidentally with an intraabdominal complication.[1] In newborns and infants these anomalies manifest as a mass, prolapsing ileal loop or discharge over the umbilicus and needs urgent surgical intervention.[2-4] In the present report we are presenting our experience of managing these anomalies in newborns and infancy in a tertiary care hospital of Northern India.

## MATERIALS AND METHODS

All patients who were diagnosed as symptomatic remnants of VID who required surgical intervention were included in the study from Jan 2009 to May 2013. Their age at presentation, mode of presentation, diagnosis, treatment done and outcome were analyzed. The case notes, operation notes and discharge summary sheets of the patients were reviewed retrospectively.

A total of 16 patients who had symptomatic vitellointestinal anomalies in neonates and infants were included. The children included 13 boys and 3 girls, with ages ranging from 4 days to 1 year (mean 2months) at presentation. Out of those, maximum (9) patients presented early i.e. within 1 month of life and rest (7) presented after 1 month of life.

## RESULTS

Among 9 patients who presented in neonatal period, 6 patients had umbilical prolapse of ileal loop and remaining 3 came with complaint of fecal discharge from umbilicus (Fig. 1). In 1 of the 6 patients with umbilical prolapse there was associated cleft palate and 3 had ileal loop prolapse with intussusception. Among 7 patients who presented late, 3 had bloody discharge as well as swelling over umbilicus, 1 had obstructed umbilical hernia (Fig. 1) and 3 had umbilical discharge. Exploratory laparotomy via either transumbilical (n=6) or infra-umbilical (n=8) approach was done in all except 2 patients; resection with anastomosis was performed for persistence of vitellointestinal duct (n=9), Meckel's diverticulum as content of hernial sac (n=1). Umbilical exploration and cyst excision was done in two cases of umbilical cyst and granuloma excision was done in 2 other cases. Two patients cannot be operated as they were hemodynamically unstable and despite resuscitation and electrolyte management they could not be stabilized. Table 1 summarizes details of the patients who underwent surgery. Histopathologically, all specimens showed normal ileal mucosa except 1, which showed ectopic gastric mucosa (Fig. 1).

**Figure:**
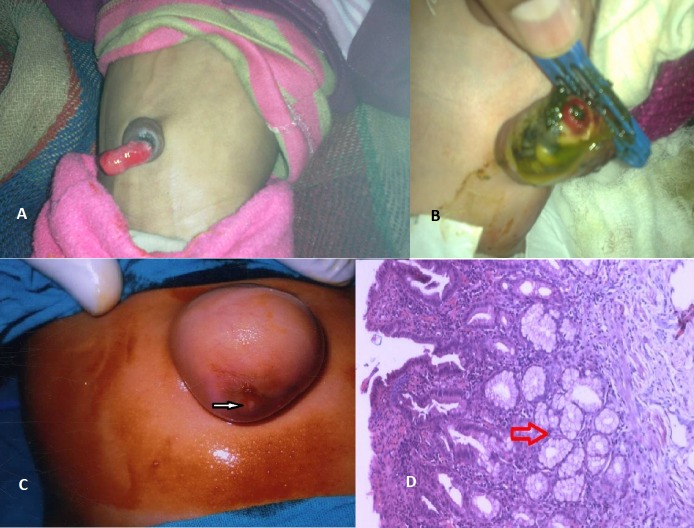
Figure 1: A- Prolapsed patent VID. B- Meconium discharge from patent VID. C- Umbilical hernia harboring MD. Arrow shows site of attachment of MD to the underside of skin. D- Histopathology showing ectopic gastric mucosa (Arrow).

**Figure:**
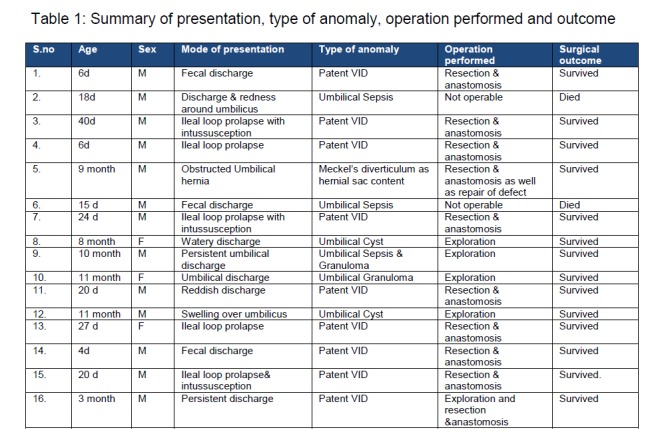
Table 1: Summary of presentation, type of anomaly, operation performed and outcome

Both the patients who could not be operated expired whereas there was no postoperative mortality. Postoperatively, wound infection occurred in 2 patients that was managed conservatively.

## DISCUSSION

OMD remnants (VID anomalies) are congenital anomalies that continue to grow if it fails to completely atrophy and disintegrate [1,4]. Although these malformations are found with equal frequency in both the sexes, a significantly greater incidence of symptoms is encountered in males.[4] Similar sex distribution was present in our study with predominance of males.

They may be asymptomatic; however, common presentations include abdominal pain, rectal bleeding, intestinal obstruction, umbilical drainage and umbilical hernia. [4-6] Similar to our experience, most of the subject are symptomatic in the neonatal period. Two of the subjects presenting in neonatal age succumbed to neonatal sepsis and dyselectrolytemia. Although MD is common OMD anomaly in children, it is rare in infancy. [5,6] In our study too only in one case MD was found.

The connection in patent VID is usually to the ileum, but less commonly to the appendix or colon.[6,7] The patient may present with the anomaly itself or due to complications like intestinal obstruction secondary to volvulus, intussusceptions or adhesions. The patients presenting with bowel prolapse along with intussusception require emergent intervention to avoid gut ischemia.

Umbilical granulomas (UG) are the commonest umbilical abnormalities encountered in neonates; they are round, moist, erythematous, pedunculated and usually between 3 and 10 mm in diameter. Bacterial colonization and low-grade infection may play a role in their pathogenesis. The common treatment is cauterization with 75% Silver Nitrate, usually repeated two to three times. Rarely, persistent UG need surgical removal. We included only 2 patients who did not recover on conservative management and later were explored. Histopathology revealed bowel mucosa indicating its origin from VID.

Other modes of presentation such as umbilical cysts or polyps are less common [8].

The treatment of a patent VID is wedge or segmental resection. In wedge resection; there are chances that ectopic mucosa may be left in native ileum, hence resection and anastomosis was our preferred approach. The prognosis is generally good except omphalitis associated with VID anomalies; especially if associated with complications has been reported to have significant mortality.[9] In this study, 2 patients with umbilical sepsis and dyselectrolytemia died; morbidity and mortality could have avoided by early diagnosis and treatment. 

In conclusion, our experience of VID anomalies revealed that they constitute a significant group of anomalies at the umbilicus in newborns and infants with varied presentations, most common being patent VID. Patients with excessive prolapse, or high output umbilical fistula should be explored early to avoid morbidity and mortality as happened in two of our patients.

## Footnotes

**Source of Support:** Nil

**Conflict of Interest:** None
